# Single-stage bilateral conversion arthroplasty for hip fusion via direct anterior approach in a patient with severe ankylosing spondylitis and kyphoscoliosis: a case report

**DOI:** 10.1186/s13256-023-04251-y

**Published:** 2023-12-11

**Authors:** Shahabaldin Beheshti Fard, Sina Afzal, Mohammadreza Barzegar, Seyed Mohammad Javad Mortazavi

**Affiliations:** 1 Joint Reconstruction Research Center, Tehran University of Medical Sciences, Tehran, Iran; 2 Department of Orthopedic Surgery, School of Medicine, Shahid Beheshti University of Medical Sciences, Tehran, Iran

**Keywords:** Ankylosing spondylitis, Kyphoscoliosis, Total hip arthroplasty, Hip, Case Report

## Abstract

**Background:**

Severe ankylosing spondylitis (AS) frequently involves hip joints and, occasionally, presents with concurrent spinal deformities, such as kyphoscoliosis, creating complex challenges for surgical management.

**Case presentation:**

We present a 26-year-old Persian male with a history of AS and severe kyphoscoliosis, leading to bilateral hip fusion and immobility. Following spinal deformity correction, a one-stage bilateral conversion to total hip arthroplasty (THA) was conducted through the direct anterior approach.

**Conclusion:**

Primary correction of spinal deformities allows for extended surgical procedures under general anesthesia. Single stage bilateral hip conversion arthroplasty via the direct anterior approach enhances postoperative mobilization, reduce the risk of re-ankylosis, and improve the overall quality of life for AS patients with this unique presentation.

## Introduction

Ankylosing spondylitis (AS) is an inflammatory rheumatic disease belonging to the group of spondylarthritis, mainly involving axial skeleton and inflammation of the joints presenting initially with inflammatory low back pain in most of the cases [1]. AS classification and diagnosis is made based on a combination of at least one clinical criteria including low back pain and stiffness, lumbar spine motion restriction, chest expansion restriction, plus radiological criteria of sacroiliitis unilaterally or bilaterally according to the Modified New York criteria 1984 for AS [2]. The series of pathologic events happening in AS process in years leads to structural changes and deformities in axial skeleton that cause major disability and altered quality of life of patients [3].

Hip involvement in AS reported to happen in about one-third to half of the cases, and in most of them this involvement is diagnosed in bilateral hip ankylosis [4]. Conversion of the hip fusion to total hip arthroplasty (THA) is the gold standard treatment of such cases that brings back the normal function to some extent and improves the quality of life of the patients [5, 6]. The results of THA in patients with AS and ankylosed hips are frequently reported [4, 5, 7, 8], and in some studies this surgical approach is used for bilateral cases to convert the fusions to arthroplasties and regain the hip function in patients [9–11].

However, simultaneous presence of AS and spine deformities like kyphoscoliosis is a relatively rare phenomenon reported in studies, with almost none of them targeted to repair the hip fusion [12–15]. In the rare case we present in this study, the patient was diagnosed with AS and severe kyphoscoliosis who was suffering from bilateral hip ankylosis that underwent single-stage bilateral hip fusion conversion to THA after spine surgery that corrected the kyphoscoliosis and let us to place the patient in supine position and perform conversion surgery in one session. The findings of this study may be beneficial in the management of similar complex cases with challenging condition making anesthesia and surgery difficult and impossible without primary correction of the spinal deformity.

## Case presentation

### History and physical examination

A 26-year-old Persian male patient was referred to our tertiary orthopedic surgery center with chief complaint of bilateral pelvic pain since a couple of months ago. The patient had a history of diagnosed AS and thoracolumbar kyphoscoliosis. The drug history of patient included only sulfasalazine 2 g/day PO divided BID for the treatment of AS. No other specific past medical history was present. Patient had no history of smoking or drug abuse. Family history was unremarkable specifically regarding the rheumatologic conditions.

**Fig. 1:**
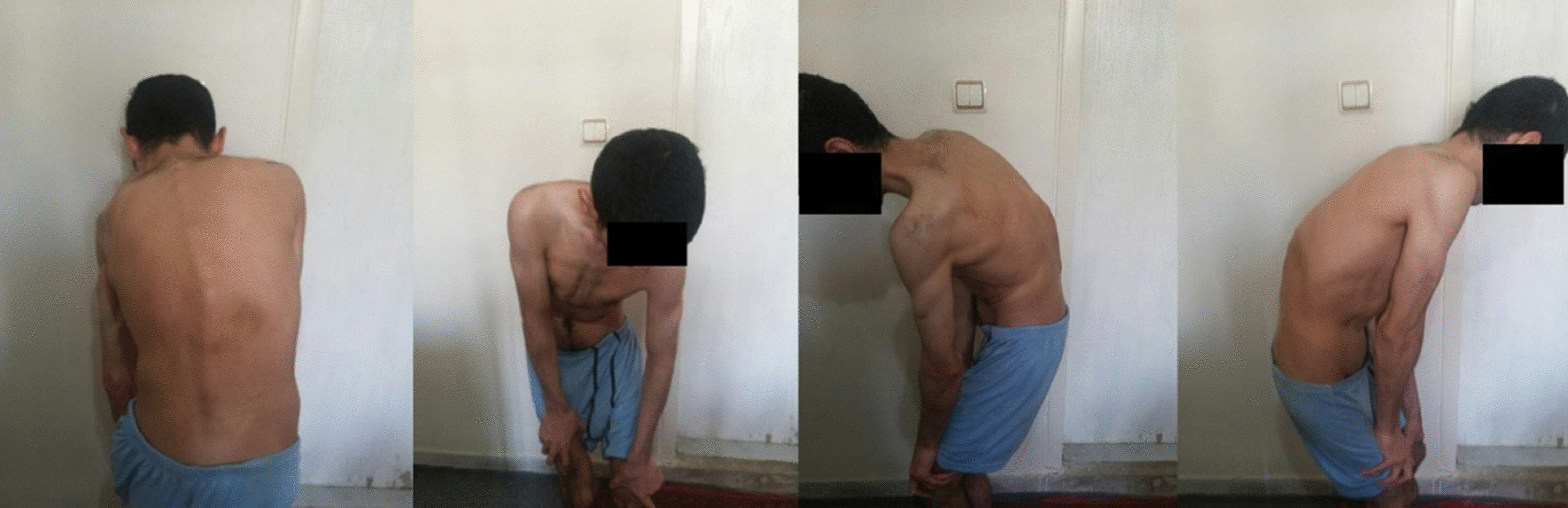
Patient presentation before the kyphoscoliosis correction surgery

On the physical examination, the patient was thin and an apparent kyphoscoliosis was observed. Also, the range of motion (ROM) of the hip joints bilaterally was severely limited that made walking almost impossible for patient. Also, the kyphoscoliosis made placing the patient in the supine position not possible in any way **(**Fig. 1**)**.

### Radiologic assessment

**Fig. 2:**
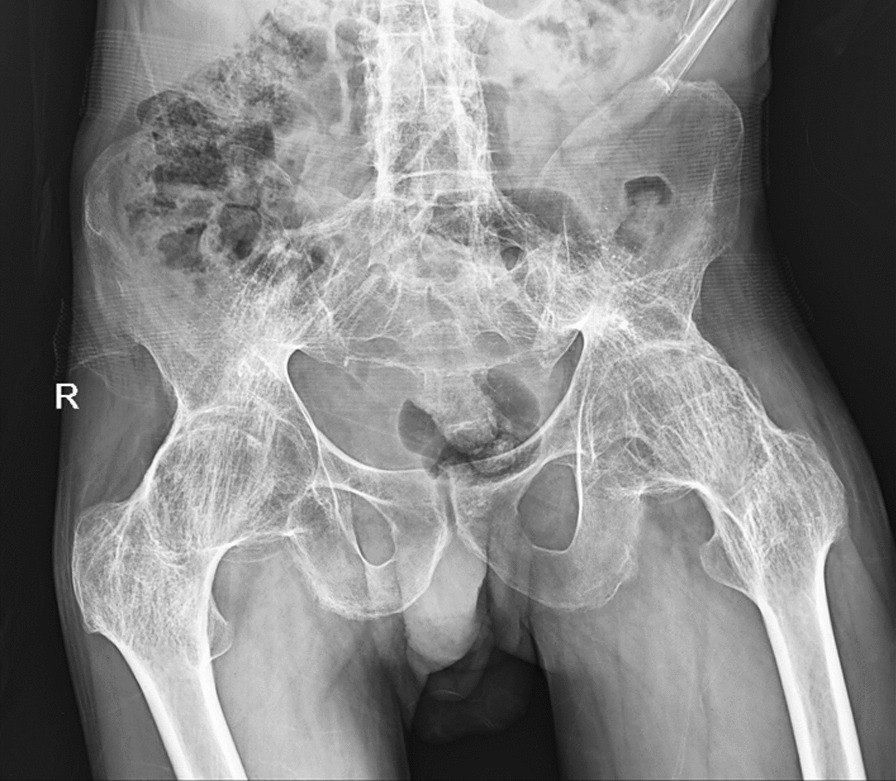
Initial pelvic plain radiograph revealed bilateral fused hips and bamboo spine

**Fig. 3:**
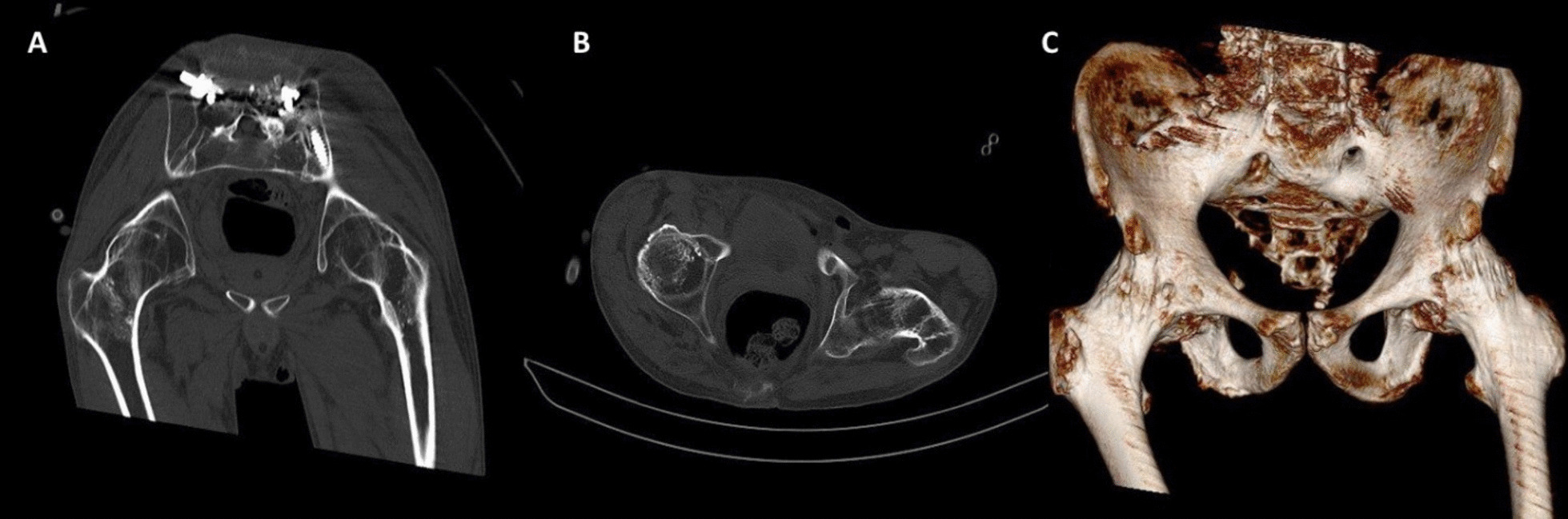
Computed tomography evaluation of the hip in **A** coronal and **B** Axial planes, and **C** with further 3 dimensional reconstruction

Pelvic X ray revealed bilateral fused hips and bamboo spine **(**Fig. 2**)**. Further evaluation with computed tomography (CT) scan and 3D reconstruction confirmed ankylosed hips in their respective anatomical locations **(**Fig. 3**).**

### Treatment plan

In according to the potential surgical and anesthetic risks associated with performing total hip arthroplasty (THA) in patients diagnosed with AS, a comprehensive consultation with the patient led to the decision to choose a one-stage conversion of bilateral hip fusion to THA. To optimize the surgical procedure, the direct anterior approach (DAA) was chosen as the preferred method. In preparation for this conversion, the patient was initially referred to a spine specialist to address the underlying spine deformity, thereby enabling the patient to assume a supine position during the THA procedure. After nine months following the spinal surgery, the patient underwent readmission for a one-stage bilateral hip conversion arthroplasty via the DAA. This approach allowed for the simultaneous performance of both hip surgeries, promoting enhanced postoperative recovery and facilitating quicker mobilization for more efficient restoration of hip joint function.

### Surgical procedure

**Fig. 4:**
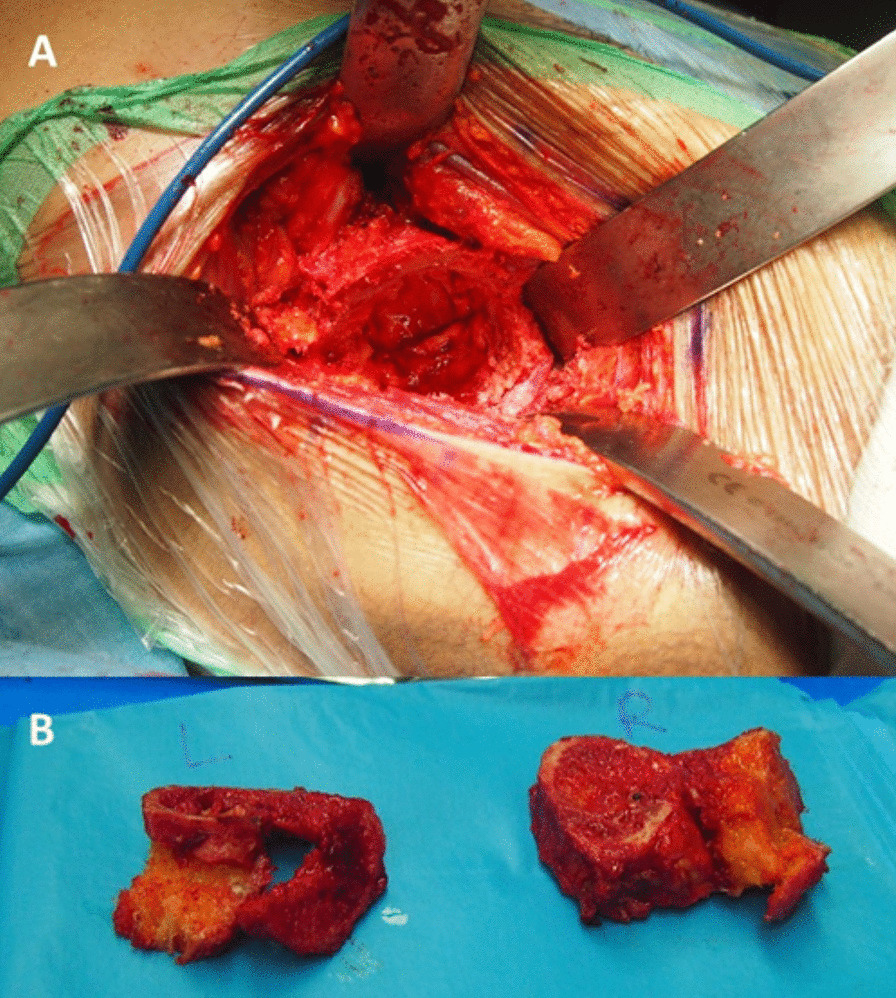
**A** intraoperative view of hip fusion conversion surgery. **B** Removed bone blocks during osteotomy of the fused hips

To perform the conversion arthroplasty, the hips were approached using the DAA in a single-stage surgery. The surgical approach involved utilizing the intermuscular plane between the Sartorius muscle and the tensor fascia lata. after reaching the joint two cuts were made utilizing an oscillating saw, one in the basicervical region (proximal to the lesser trochanter), and the other in the subcapital region (the confluence of the neck and ileum) **(**Fig. 4**)**.

**Fig. 5:**
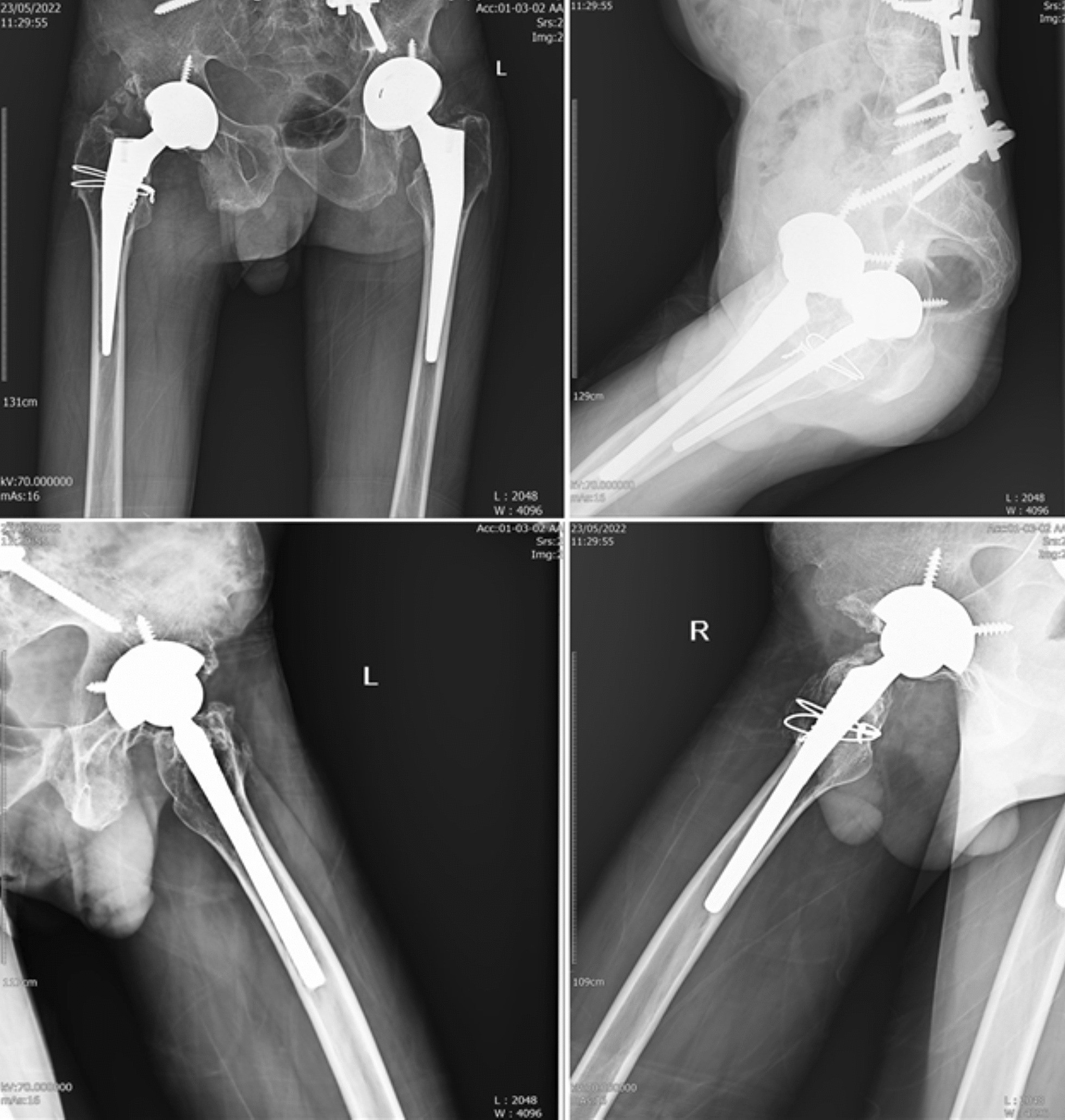
Post-operative plain radiograph revealing the placed arthroplasty and devices

After the removal of the bone block, we proceeded with the surgical placement of the acetabular cup and femoral stem with precision. During acetabular reaming, particular attention was paid to ensure the correct version, inclination, and medialization of the components. Given the significant muscle atrophy observed in this case, we assessed the feasibility of using a larger cup size to accommodate a larger femoral head. Post-operative radiographic assessments confirmed the proper anatomical positioning and version of the prosthesis components **(**Fig. 5**)**.

### Postoperative care and follow-up

The patient was suggested to walk using a pair of crutches for a duration of two months, while maintaining full weight bearing. Also, the patient was warned about hip deep flexion with internal rotation, and hyperextension with external rotation of the hip for six months after surgery to reduce the possibility of dislocation.

Patient was followed up for 12 months. He started to walk independently and acceptably at the end of the follow-up period and experienced an uneventful period after surgery.

## Discussion

This study shows the surgical management of a challenging case involving ankylosed hips on both sides in a patient with AS and kyphoscoliosis. The patient underwent a successful single-stage surgery to convert both hip fusions using the DAA approach. The results have been promising during the follow-up period and are encouraging for the long-term prognosis.

Anesthesia administration in AS cases poses significant challenges, primarily attributed to limitations in patient positioning caused by severe axial skeleton stiffness and spinal rigidity. These factors not only hinder patient mobility but also restrict respiratory capacity [16, 17]. This issue is much more prominent in cases with AS and accompanying spine deformities like kyphoscoliosis that multiplies the challenges due to cervical spine and temporomandibular joint mobility limitations and further restrictions in respiratory capacity as in these complex cases the thoracolumbar spine is severely affected and deformed [12, 18].

In our search of literature, only a few studies have reported performing surgeries on such cases and none of them were on surgical repair of fused hips due to AS. Here we review the presentations and findings of those cases and studies to provide a bigger picture of the patient management in these cases.

In a study by Kuwaijo and colleagues on a 58-year-old man with AS and severe kyphoscoliosis who was diagnosed with prostate carcinoma, the surgeons placed the patient under general anesthesia in the supine-Trendelenburg-Lithotomy position and performed the robot-assisted radical prostatectomy and this way they could overcome the complex state of the patient and the surgery was successful [19]. The second case was a 73-year-old man with AS and severe thoracolumbar kyphoscoliosis diagnosed with benign prostatic hyperplasia and was a candidate for transurethral resection of prostate under regional anesthesia; however, due to failure of epidural anesthesia because of spine deformity, and the team decided to use low-dose esketamine combined with sevoflurane inhalation to anesthetize patient to perform the intervention and they were successful [12]. Review of these two cases show the difficulty of any surgery even non-orthopedic and not on the fused hips in these complicated cases.

In a published series of four cases with AS complicated by congenital spinal deformity, with one case having lumbar kyphosis, one case having thoracic kyphosis, one case having decreased L5 vertebral body height, and one case having thoracolumbar kyphoscoliosis, only one patient underwent poly-segmental osteotomy and fixation of spine and the others were managed by only medications, and none of the cases has evidence of hip involvement at the stage of their presentation with lower back pain and stiffness [14]. Another case series reported 25 consecutive patients with AS-related thoracolumbar kyphosis with different degrees of spine and hip deformity who all underwent surgery for spinal deformity correction, they reported that the main challenges against performing surgeries in these patients are a combination of cervical stiffness, coronal imbalance, and hip involvement that necessitate individual surgical planning for each case [13]. Also, that study reported a high rate of post-operative complication in more than 50% of the operated cases that were timely captured and cured [13].

Speaking of complications, evidence in the filed show that AS patients with and without spinal deformities are at higher risk of post-operative adverse effects and complications which need appropriate consideration and management. A systematic review of available literature on the bilateral THA conversion surgery in AS patients found that this treatment is safe and effective in repair of hip fusion in these cases; however, the technical obstacles exist in their surgery and the risk of post-operative complications are significant including intra-operative fracture, nerve injuries leading to paresis and palsy, hip dislocation, the need for revision surgery mainly due to aseptic loosening, and the serious issue of development of heterotopic ossification after surgery and the risk of re-ankylosis if the primary disease of AS is not well under control [11]. Other intra-operative complications are probable damages to adjacent elements like proximal femur or spinal fracture which later has double significance in cases with AS and spinal deformity at the same time which might further complicate the disease management [20].

Performing the single-stage bilateral conversion THA surgery via DAA in the case presented in this study was a strength of our approach which enabled earlier patient mobilization and rehabilitation. Studies show bilateral THA for severe hip ankylosis in AS cases is an efficient method to regain the hip joint function, relieve the pain, and improve the patient quality of life [9]. Another investigation that followed a series of 19 cases with bilateral hip ankylosis due to AS for a mean period of 82.5 months and reported the mid-term results found that outcomes in a relatively long run was also satisfactory, and most of the minor complications resolved after a while and no major complication like hip dislocation, infection, and deep vein thrombosis was detected in their followed cases [10].

## Conclusion

AS complicated by spinal deformities like kyphoscoliosis is a challenging situation for any surgery, specifically in case of hip involvement in the form of ankylosis and hip fusion, as spinal deformity poses a major obstacle against anesthesia and essential maneuvers during surgery. A primary correction of the spinal deformity provides the chance for performing longer surgeries under general anesthesia and a single-stage bilateral hip fusion conversion to THA facilitates patient mobilization and recovery. Also, performing the conversion arthroplasty via the direct anterior approach brings up the chance of hip conversion in both sides in a single surgery so the patient mobilization could be quicker. Choosing a proper treatment plan for patients with simultaneous AS and kyphoscoliosis needs a by-case decision making process and approach to reach the maximum benefits for the improvement of patient’s quality of life.

## Data Availability

Not applicable.

## Abbreviation

AS: Ankylosing spondylitis
THA: Total hip arthroplasty
ROM: Range of motion
CT: Computed tomography
DAA: Direct anterior approach
